# 2237 From bedside to benchmarks: A physician-scientist workforce dashboard for biomedical research institutions

**DOI:** 10.1017/cts.2018.214

**Published:** 2018-11-21

**Authors:** Adrienne Zell, Lindsey Smith, David Yanez, Jeanne-Marie Guise, David Ellison

**Affiliations:** Oregon Health & Science University

## Abstract

OBJECTIVES/SPECIFIC AIMS: A growing concern about the declining physician-scientist workforce prompted the 2014 National Institutes of Health (NIH) Physician Scientist Workforce to recommended that “tools for assessing the strength of the biomedical workforce” be developed. To aid strategic planning, the Oregon Clinical and Translational Research Institute convened key stakeholders at its home university, Oregon Health and Science University (OHSU), to survey the local landscape of physician scientists. Surprisingly, few consensus methods were available to measure and benchmark OHSU with respect to national comparators. To address this deficit, we sought to develop clear and objective metrics describing physician-scientist success at our institution. By focusing on local funding, we were able to generate more complete and robust data than others have reported. These data also permit us to compare ourselves to the national workforce, using well-curated and accessible national databases. The goal of the analyses is to contribute to strategic decision-making by portraying the local physician-scientist workforce, comparing it to the national landscape, and making recommendations about mechanisms to address potential opportunities. This has led us to develop a simple quantitative dashboard, which now permits OHSU to craft strategic targets and address successes and opportunities. These approaches are likely to be valuable elsewhere. METHODS/STUDY POPULATION: OHSU is a medium-sized academic health center in Portland, Oregon with over 1200 principal investigators and over $230M in NIH funding. The primary focus of our investigation was physician-scientists who receive extramural funding. To align with other analyses, we distinguish physician-scientists with an M.D. only, or with an M.D. and a master’s degree, from physician-scientists who hold an M.D./Ph.D. For this distinction, we use the indicator “M.D.-only” to indicate the former. The study design consisted of (a) selection of available and relevant national level data on the physician-scientist workforce, (b) curating of local level data to align it with the national indicators, (c) comparing the 2 sets of data to look for differences in trends over time, and (d) supplementing the analyses with additional local data not available at the national level. Key comparisons were tested for statistical significance and plotted on a dashboard, which was then reviewed by an OHSU internal working group focused on physician-scientists. Data elements included degrees, age, gender, and grants awarded. National data come directly from the NIH Data Book, updated for fiscal year 2016. The NIH makes all funded project data available in the publicly downloadable ExPORTER Data Catalog. These project data were used to supplement the summarized data available from the NIH Data Book, allowing us to extract OHSU investigators and to complete the K to R comparative analysis. For analyses of OHSU investigators holding funding other than RPGs, we relied on institutional data from the OHSU grants and contracts office. Demographic data on OHSU investigators were obtained from departmental and human resource records. The time period for these analyses was 1998–2016. RESULTS/ANTICIPATED RESULTS: At OHSU, as nationally, there has been an increase in RPG-holding Ph.D.s but not in RPG-holding physician-scientists. At OHSU, nearly three-fourth of physician-scientist RPGs hold an M.D.-only degree, compared with nationally, where nearly half of physician-scientists are M.D./Ph.D.s. The percent of younger, early-career, RPG-holding physician-scientists has declined precipitously at OHSU and nationally. At OHSU, the percentage of RPGs held by women physician-scientists is below the national figure. Funding sources for physician-scientists at OHSU were more diverse than for Ph.D. scientists, and physician-scientists comprise the majority of Principal Investigators on clinical trials. These non-RPG sources of funding remain a critical source of support, although local analyses of time spent on research indicate that physician-scientists with NIH funding spend a greater percentage of their time on research than those without. OHSU PI’s have had success in transitioning from K08 and K23 grants to R-level grants, with similar percentages receiving RPGs within 5 years. A dashboard comparing these trends was developed. DISCUSSION/SIGNIFICANCE OF IMPACT: There were 3 key impacts from our analyses. First, we developed and disseminated a dashboard with both local data and national comparators. Second, in consultation with institutional leadership, we selected target values to define success for each metric. Third, we recommended actions that will help OHSU meet the selected targets. A major accomplishment of this structured approach has been the identification of opportunities for change that were not recognized previously. For example, leadership was not aware of the substantial and growing deficit in female physician-scientists at OHSU, compared with the impressive increases nationally. Thus, to reduce gender disparity at OHSU, we have recommended purposeful recruitment; one approach is to target female graduates of Medical Scientist Training Programs for faculty positions, as this group has better success at achieving R-level funding than do M.D.-only applicants. Another outcome is to help set ambitious but reasonable targets for improving the local landscape. Thus, we aim to reduce the average age of RGP-holding physician-scientists at OHSU by one year during the next 5 years. Although reversing current trends will not be easy, our analyses suggest that the average age of RPG level physician-scientists at OHSU would decrease were OHSU were to match the national-level proportions of women and M.D./Ph.D. physician-scientists. In addition to targeting gender disparities, we have recently implemented a program that supplements funding for recruiting young physician scientists, and then supporting their pursuit of RPG funding. Locally, a bright spot is the K to RPG transition rate for K23 awardees, which compare favorably with national data, an outcome that we plan to maintain. In analyzing this area of success, one reason is our strong mentorship program, called OCTRI Scholars, which is provided through our CTSA-sponsored institute. This has fostered an atmosphere of success among young physician-scientists and is one of the reasons that we endorse recommendation #9 from the PSWR, suggesting that Clinical and Translational Science Award (CTSA) Institutes play pivotal roles in monitoring and enhancing the success of the physician-scientist workforce. Thus, several perceived deficiencies might be addressed with adjustment of 1 or 2 specific institutional policies. While the specific opportunities and strengths may be different at other institutions, our proposed dashboard, which couples publicly curated, freely accessible databases, with readily available institutional resources, should help institutions to set and achieve their own goals.

